# A Glass-Fiber-Optic Turbidity Sensor for Real-Time In Situ Water Quality Monitoring

**DOI:** 10.3390/s23167271

**Published:** 2023-08-19

**Authors:** Chi Thanh Vu, Amir Ahmadi Zahrani, Lingze Duan, Tingting Wu

**Affiliations:** 1 Department of Civil and Environmental Engineering, University of Alabama in Huntsville, Huntsville, AL 35899, USA; 2 Department of Physics and Astronomy, University of Alabama in Huntsville, Huntsville, AL 35899, USA

**Keywords:** optical sensors, fiber optics, turbidity measurement, drinking water, real-time detection

## Abstract

Turbidity is an important water quality parameter, especially for drinking water. The ability to actively monitor the turbidity level of drinking water distribution systems is of critical importance to the safety and wellbeing of the public. Traditional turbidity monitoring methods involve the manual collection of water samples at set locations and times followed by laboratory analysis, which are labor intensive and time consuming. Fiber-optic measurement permits real-time, in situ turbidity monitoring. But the current technology is based on plastic fibers, which suffer from high optical attenuation and hence are unsuitable for large-scale remote monitoring. In this paper, we report the demonstration of a fiber-optic turbidity sensor based on multi-mode glass fibers. The system uses a single fiber to both deliver laser light into the water sample and collect the back-scattered light for detection. A balanced detection scheme is utilized to remove the common-mode noise to enhance the turbidity sensitivity. Highly linear turbidity responses are obtained and a turbidity resolution as low as 0.1 NTU is achieved. The test unit is also shown to have excellent reproducibility against repeated measurements and good stability against temperature changes. Turbidity measurement in real environmental matrices such as tap water and pond water is also reported with an assessment of the impact of flow rate. This work demonstrates the feasibility of future large-scale distributed fiber-optic turbidity monitoring networks.

## 1. Introduction

Turbidity is caused by the presence of suspended particles, organic matter, and chemicals, and is widely measured in natural resources, irrigation water, the food and beverage industry, and drinking water [1,2,3]. As an important water quality parameter, turbidity not only indicates the efficiency of some treatment processes (e.g., sand filtration) but also reflects water quality changes in the distribution systems. For example, cast iron and steel pipes constitute a large proportion of drinking water distribution systems (DWDS) in many countries (e.g., 57% in the USA [4]), where the internal corrosion is ubiquitous [5]. Disturbance of corrosion scale due to changes in water quality or hydrodynamic conditions may result in water discoloration and/or contaminant release, leading to consumer complaints and a potential threat to public health. Discolored water episodes (red water) have been reported worldwide such as in the U.S. [6,7], China [5], and European countries [8,9]. Turbidity measurement, particularly the continuous monitoring at several locations at the same time, has been suggested as a practicable technology providing data to be used to identify causal factors and quantify discoloration risks [10]. Moreover, turbidity has been correlated with contamination with Giardia and Cryptosporidium and used as a surrogate measure for the risk of contamination by these pathogens [11]. Studies also revealed a strong temporal relationship between turbidity and gastrointestinal events during and preceding the major waterborne disease outbreak in Milwaukee in 1993 [12]. All these findings emphasize the importance and necessity of turbidity monitoring in a contamination warning system, preferably with cost-effective and real-time monitoring methods.

Turbidity can be measured either by determining the degree of light transmission (turbidimetry) or by evaluating the degree of light-scattering (nephelometry) [13]. The major standard methods include the US EPA method 180.1, ISO 7027, and the GLI method 2 [14,15,16]. In practice, turbidity can be measured using a turbidity instrument in the lab or a portable turbidimeter in the field. Several on-line, reagent-free water monitoring systems are also commercially available, but the bulky size and high cost prohibit their application in DWDS [17,18]. Solid-state multi-parametric sensor arrays incorporating turbidity sensors have also been developed [19]. Further, research efforts have been taken to design smart sensor networks. For example, low-cost water quality sensor nodes which consist of sensing, data-processing, and communicating components have been investigated. In these, the measurement nodes that are interfaced to multi-parametric sensor arrays are proposed to be installed in a spatially distributed manner and form a wireless sensor network [18,19,20,21,22]. While the networking capability is appealing, both the hardware and the software of such systems need further improvement. In addition, the sensing performance and the lifetime may be limited by the battery power, leading to extra maintenance costs [20].

Innovative design of the turbidimeter is vital to achieving accurate measurements as well as to develop robust and low-cost distributed sensors. In this regard, fiber-optical turbidity sensors possess some important advantages such as low cost, compactness, great flexibility, high stability over a wide temperature range, immunity to electromagnetic interference, water and corrosion resistance, and compatibility with multi-sensor schemes [23,24,25]. An optical fiber turbidity sensory system generally consists of a light source, a sensing element (transducer), a detector, and optical fibers which act as a light transmission medium between water samples and the receiver circuit. Often, one or more fibers are used for emitting light and the rest are used for receiving the light reflected/scattered from the water sample [26,27,28,29]. Although the potential applications of optical fiber-based turbidimeters in remote sensing and multi-sensor systems have been acknowledged, research on developing such sensor networks is very limited. Most previous studies used plastic optical fibers with a core diameter of 1 mm [23,24,25,26,27,28]. While they are more flexible and rugged as well as easier to handle and install than glass fibers, plastic fibers suffer from very high optical attenuations, which limits the typical fiber lengths to below 100 m. This sets the requirement for the interrogation and detection system to remain “local” to the water source, inherently prohibiting remote, off-site turbidity measurement. Meanwhile, future DWDS call for large-scale, distributed turbidity-monitoring networks for real-time, in situ drinking water quality monitoring. There is hence a demand for turbidity sensors based on low-loss glass fibers.

In an effort to address the aforementioned challenges, in this study, we designed and developed an innovative, low-cost glass optical fiber-based turbidity sensor as the foundation for future development of real-time, in situ sensor networks. The turbidity sensing properties of the sensor were systematically evaluated in the laboratory. The performance was examined with real environmental samples under the influence of temperature and flow rate to verify the feasibility of turbidity measurements for the proposed applications.

## 2. Methods

### 2.1. Design and Measurement Principle

Turbidity represents the optical clarity of water, which can be measured by an angular distribution of scattered light (i.e., nephelometry) or a reduction in the intensity of transmitted light. In this study, back-scattering is used to determine turbidity to circumvent the difficulties of transmitted light measurement in low-turbidity samples and interference by light absorption caused by dissolved species in the water samples. Moreover, compared to other nephelometry approaches, the back-scattering approach does not require separate light transmitters and receivers. A single fiber can simultaneously deliver light into the water and collect the back-scattered light, allowing an easy scale-up of the number of probes and, hence, making a distributed scheme possible.

The main technical challenge for a glass-fiber-based nephelometer is the small core sizes of the glass fibers. Typical core sizes of glass fibers range from 10 µm to 200 µm in diameter, which are markedly smaller than the core sizes of plastic fibers (typically > 1 mm). As a result, the amount of scattered light that can be collected by a glass fiber is orders of magnitude lower than a plastic fiber due to the much smaller light-collecting area of the glass fiber. With lower collected optical power, glass-fiber turbidity sensors are projected to have poor signal-to-noise ratios (SNR) and hence reduced turbidity sensitivity. This is why plastic fibers have been widely studied for turbidity measurement while little research has been reported on glass-fiber-based nephelometers.

To overcome this shortcoming of glass fiber, we propose here a balanced photodetection scheme, where the use of differential detection helps remove much of the common-mode noise (e.g., laser intensity fluctuation, fiber-end reflection, etc.). This allows a large electronic gain to be utilized to counter the small collected optical signal from scattering. The scheme has shown great promise to measure turbidity down to the levels relevant to drinking water, as described in the following sections.

### 2.2. Experimental System, Materials and Equipment

**Figure 1:**
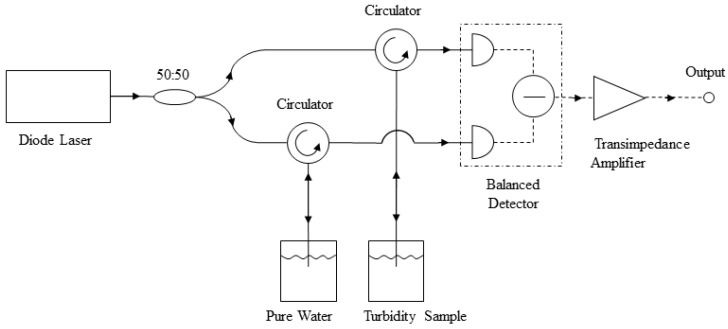
The conceptual layout of the glass-fiber turbidity measurement unit.

A system layout of the glass-fiber turbidity measurement unit is shown in Figure 1. The entire unit was constructed using glass fibers with a 200-µm core size and a 0.22 numerical aperture. The laser operated at 980 nm, with about 10 mW of power delivered into the water. A 50:50 fiber coupler evenly split the laser output into two arms. In each arm, a circulator routed the laser light into the water samples. Two water samples were used for measurement, one pure water sample as the reference and one “polluted” sample for turbidity determination. Such an arrangement allowed the large fiber-end reflection to be subtracted off via the detector. The back-scattered light from the samples was collected by the same fibers and was directed toward the detector by the circulators. A balanced Si photodetector (Thorlabs PDB450A) converted the optical signals into electrical signals, subtracted them to generate a differential output, and amplified this differential signal with a transimpedance amplifier (TIA). The TIA has a tunable gain that can be set between 10^3^ and 10^7^. A digital multimeter (RIGOL DM3058E) as well as an oscilloscope were used to record and monitor the TIA output.

**Figure 2:**
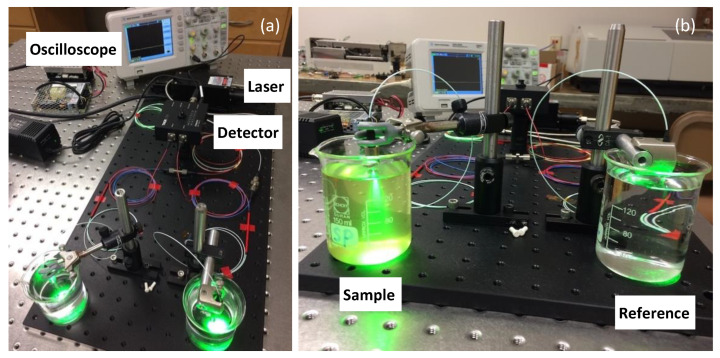
(**a**) The glass-fiber turbidity-sensing test unit developed in this study. (**b**) A comparison of the optical scattering produced by a high-turbidity sample (left) and by pure water (right).

Pictures of the actual setup are shown in Figure 2. The unit has a footprint of 1 ft. × 2 ft, with ample room for further minimization. To better illustrate the effect of scattering caused by turbidity, the laser shown in the picture operated at 532 nm. Actual measurements were performed with a more stable 980 nm diode laser. This choice of wavelength was based on the availability of stable lasers. In real applications, other wavelengths (e.g., 1.3 or 1.5 µm) may be better options for lower fiber loss.

Turbidity standard solutions (0~100 NTU) were purchased from ThermoFisher Scientific. Tap water and surface water samples were taken from the water quality lab and a pond located on the university campus. A commercial turbidity meter (Orion™ AQUAfast AQ3010) was also used to measure the sample turbidity for comparison purposes when needed. The entire experiment was conducted under normal laboratory conditions.

## 3. Results and Discussion

### 3.1. Sensor Characterization

**Figure 3:**
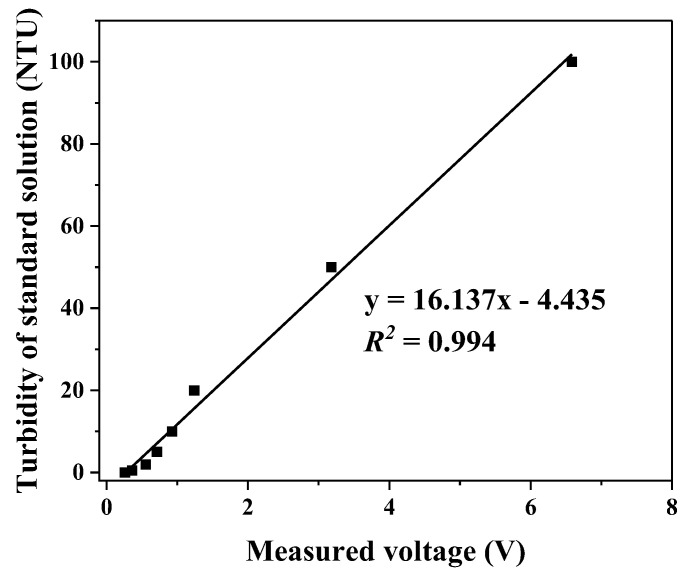
Calibration results of the glass-fiber turbidity sensor.

In order to characterize and evaluate the sensing properties for turbidity, the test unit was first calibrated with standard turbidity solutions (0, 0.5, 1, 2, 5, 10, 20, 50, and 100 NTU). Specifically, the standard solutions were used as the “sample” water and the detector outputs (voltage) were recorded. This process allowed us to establish a correlation between the sensor output voltage and the turbidity of the “sample” water. A calibration curve was successfully created between 0–100 NTU as shown in Figure 3. The excellent linearity (R^2^ > 0.99) demonstrated the feasibility of glass-fiber-based turbidity sensors. Moreover, the fact that the sensor was able to measure a turbidity level below 1 NTU showcases its potential to monitor water quality under drinking water-relevant conditions. Currently, the accuracy of the sensor in the low-turbidity region (<1 NTU) is limited by the lack of more standard turbidity solutions below 1 NTU. With better calibration capabilities, we expect much improvement in the accuracy of drinking water measurement.

**Figure 4:**
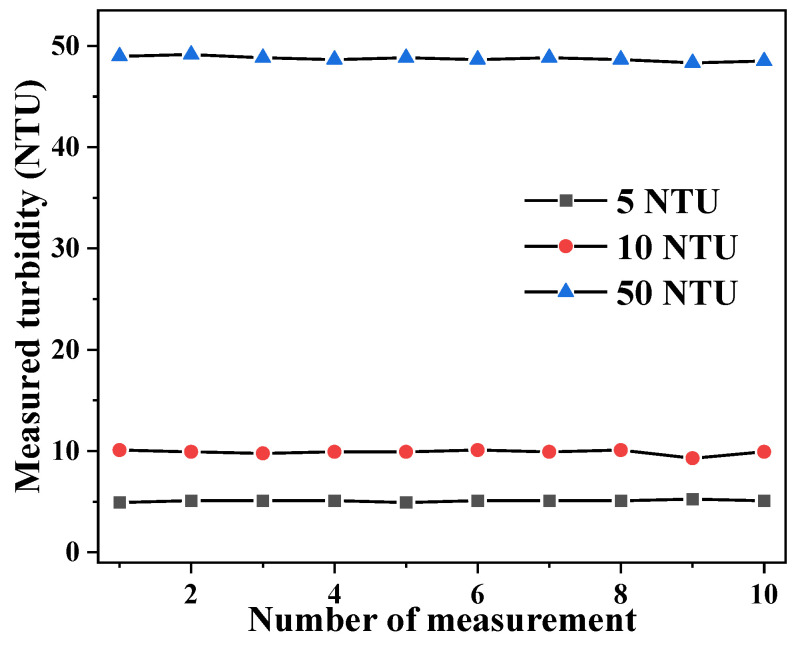
Reproducibility of the measured turbidity at 5, 10, and 50 NTU.

Next, the repeatability/reproducibility of the sensor was evaluated by measuring the same standard solution repeatedly 10 times at ~2 min intervals, where the turbidity was determined according to the calibration relation shown in Figure 3. Figure 4 shows the results of these measurements with standard turbidity solutions of 5 NTU, 10 NTU, and 50 NTU. The coefficient of variation (CV) was found to be 1.7%, 2.3%, and 0.5% for 5 NTU, 10 NTU, and 50 NTU, respectively, indicating a high degree of reproducibility of the measurement results. In each case, the fluctuation of the measured turbidity is on the order of 0.1 NTU, suggesting a noise-limited turbidity resolution of about 0.1 NTU.

**Figure 5:**
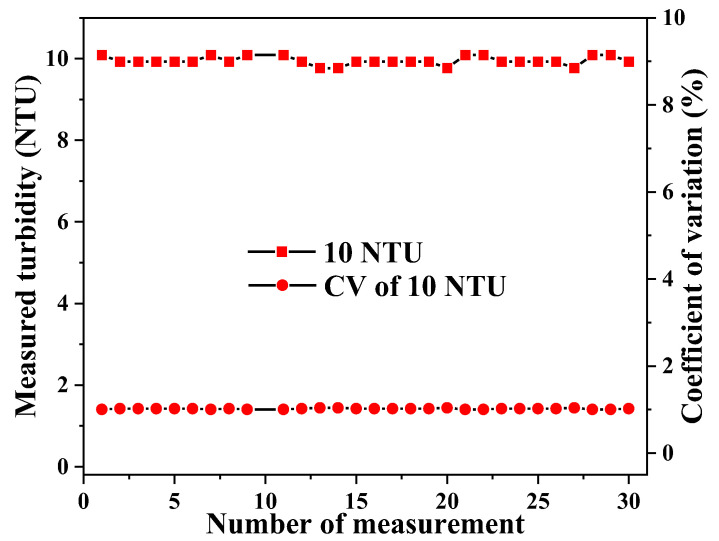
Stability of the turbidity measurement within 5 s after the sensor was dipped into a standard (10 NTU) solution.

Stability of the sensor was assessed by measuring the same standard solution (10 NTU) repeatedly 29 times. In particular, the sensor was dipped into the standard solution at a 10 s interval and the readout was recorded in 5 s. As can be seen in Figure 5, there was no significant change of the measured turbidity and the CV was ~1% for all measurements. These results demonstrate that the test unit can produce a stable output value within 5 s after the sensor is in contact with the water sample.

### 3.2. Effects of Temperature

**Figure 6:**
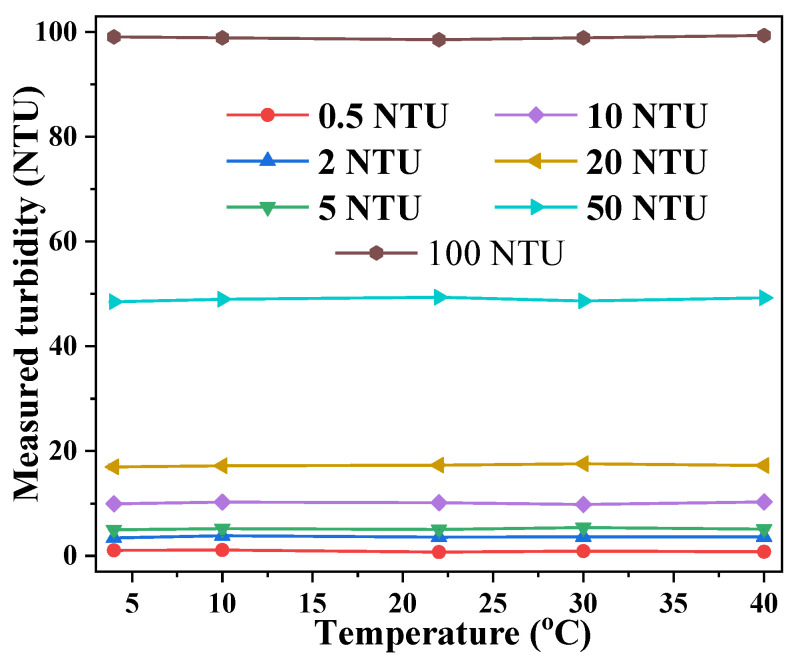
The effect of water temperature on the measured turbidity.

Since water distribution networks are subject to seasonal temperature variations, it is necessary to examine the consistency of the sensor measurement at different temperatures. Here, samples of the standard turbidity solution were cooled to 4 °C, and then gradually heated up to 40 °C. Turbidity measurement with the sensor was taken at several temperatures: 4 °C, 10 °C, 22 °C, 30 °C, and 40 °C. The measurement results are summarized in Figure 6. No significant temperature-dependent effect was observed in the sensor response within the temperature range of 4–40 °C, indicating a robust sensing scheme against seasonal water temperature variations.

### 3.3. Demonstration in Real Environmental Matrices

Lastly, to demonstrate the applicability of the glass-fiber turbidity sensor under realistic field conditions, the sensor performance was evaluated using real environmental water samples, i.e., tap water and pond water, at different flow rates (velocities). The flow velocities were selected based on the possible flow regime in water distribution networks. The characteristics of the tap water and the pond water are summarized in Table 1, where the turbidity was measured using a lab turbidity meter (static sample). It should be noted that the tap water turbidity was 0.07 NTU, which was lower than the limit of quantification of the glass-fiber sensor (~0.10 NTU).

**Figure 7:**
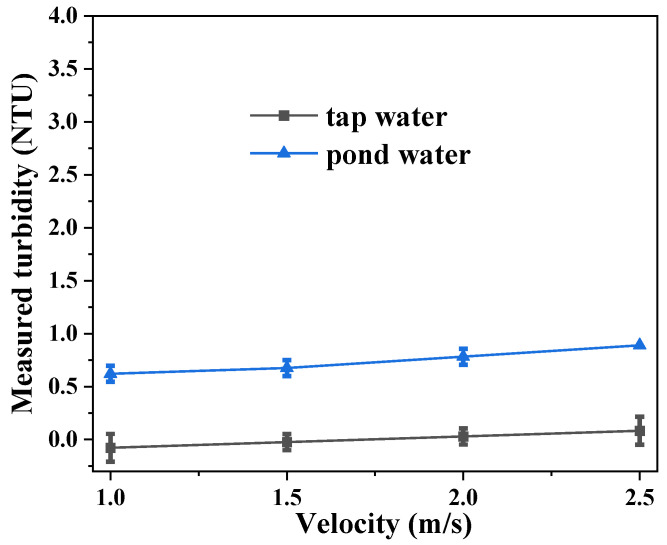
Modest increases in the sensor readings were observed as the water velocity increased.

The impact of flow rate on turbidity measurement is shown in Figure 7. Modest increases in the sensor readings, ~0.1 NTU for tap water and ~0.2 NTU for pond water, were observed as the water velocity was raised from 1.0 m/s to 2.5 m/s. Evidently, the turbulence caused by higher flow rates increases the scattering of light. In field applications, such an effect should be considered and calibrated for in order to obtain accurate turbidity readings.

It should be mentioned that the effects of soluble chemicals (e.g., chlorine and salts) on turbidity measurement were not studied in the current work. In principle, soluble contents in water can play a role in our scheme as they affect the refraction index of water. However, since most application scenarios only require the sensor to operate under a known and stable amount of soluble contents, any impact caused by these chemicals can in principle be calibrated out.

## 4. Conclusions

In conclusion, we report here the demonstration of a fiber-optic turbidity sensor based on multi-mode glass fibers. The test unit is simple and compact, using a single fiber to both deliver laser light into the water samples and collect the back-scattered light for detection. A balanced detection scheme was used to remove the common-mode noise so that large transimpedance amplifications could be employed to overcome the weak optical signal due to the smaller core sizes of the glass fibers. A highly linear turbidity calibration relation was obtained and a turbidity resolution of about 0.1 NTU was achieved. The test unit also demonstrated excellent reproducibility and stability against repeated measurements and temperature changes. The turbidity measurement was also performed in real environmental matrices such as tap water and pond water. The impact of flow rate on turbidity measurement was also assessed. It is our hope that this work can lay down the foundation for future large-scale distributed fiber-optic turbidity monitoring networks.

## Figures and Tables

**Table 1:** Characteristics of real water samples. The turbidity values were measured with a commercial turbidity meter (Orion™ AQUAfast AQ3010).

	Tap Water		Pond Water	
	Mean	STD	Mean	STD
Cl^−^ (mg L^−1^)	9.59	0.16	4.33	0.035
NO_3_^−^ (mg L^−1^)	1.05	0.005	3.95	0.025
SO_4_^2−^ (mg L^−1^)	27.1	0.025	6.26	0.035
HCO_3_^−^ (mg L^−1^)	74.4	1.2	184.2	1.2
PO_4_^3−^ (mg L^−1^)	0.44	0.015	0.16	0.005
TDS (mg L^−1^)	113.9	0.1	159.2	0.8
Conductivity (mS cm^−1^)	0.26	0.037	0.32	0.0005
DO (mg L^−1^)	6.08	0.015	6.17	0.005
pH	7.32	0.015	8.21	0.01
TOC (mg L^−1^)	3.20	0.1535	1.63	0.376
**Turbidity (NTU)**	**0.073**	**0.0047**	**0.63**	**0.014**

## Data Availability

No new data were created or analyzed in this study. Data sharing is not applicable to this article.
